# Relaxation of Japan's clozapine monitoring system is urgently needed: Adding 10,000 future prescriptions may prevent up to 400 schizophrenia‐related suicides and cost 1 death by agranulocytosis

**DOI:** 10.1002/pcn5.70357

**Published:** 2026-06-05

**Authors:** Jose de Leon, Yuki Kikuchi, Norio Yasui‐Furukori

**Affiliations:** ^1^ Mental Health Research Center, Eastern State Hospital Lexington Kentucky USA; ^2^ Biomedical Research Centre in Mental Health Net (CIBERSAM), Santiago Apóstol Hospital University of the Basque Country Vitoria Spain; ^3^ Department of Psychiatry, Graduate School of Medicine Tohoku University Sendai Miyagi Japan; ^4^ Department of Psychiatry Kodama Hospital Ishinomaki Miyagi Japan; ^5^ Department of Psychiatry, School of Medicine Dokkyo Medical University Tochigi Japan

The Food and Drug Administration (FDA) approved the use of clozapine in the United States for treatment‐refractory schizophrenia (TRS) in 1989 and for suicide in schizophrenia psychosis in 2002.^1^ Clozapine was approved with a (1) boxed warning for severe neutropenia, (2) complex hematological monitoring system, and (3) centralized database.

The FDA must review pharmacovigilance data to ensure that boxed warnings correspond to fatal outcome rates.^2^ In 1997, a company study reviewed 396 US deaths of patients ages 10–54 years during 1991–1993. The 396 deaths were classified as (1) 196 current (on clozapine or within 14 days of discontinuation); (2) 89 recent (15–106 days), and (3) 111 past (>106 days). Severe neutropenia explained only 3 (1.5%) of the 196 current deaths. Suicide explained 24 (12%) of the 196 current deaths, 18 (20%) of the 89 recent deaths, and 33 (30%) of the 111 past deaths.^3^ Recent discontinuation increased death by suicide 69% in ages 10–54 years and by 72% in those older than 54 years of age.^3^ The FDA did not appear to pay attention to this pharmacovigilance data but finally, in a November 2024 meeting, the FDA presented data compatible with an extremely low agranulocytosis mortality rate both worldwide and in the United States.^2^ Worldwide, severe neutropenia explained 3% of deaths from January 2015 to September 2023. In the United States, 2 (0.03%) deaths associated with severe neutropenia occurred in a cohort of 6488 patients followed for up to 23 years. In June 2025, the US package insert was modified to eliminate the centralized database, and hematological monitoring was redefined as “recommended.”^2^

The first clozapine application to Japan's drug agency was retracted in 1975 due to concern about agranulocytosis.^4^ During the 1990s, several lawsuits against the Japanese drug agency concerning drug‐related deaths led to slower approval of new drugs. Thus, many psychiatric drugs that had been approved in Western countries took many years to be approved in Japan. The next clozapine application took from December 2007 to April 2009 for approval.^4^ Moreover, the use of clozapine in Japan is different from the rest of the world.^5^ In the rest of the world, clozapine is generic, but in Japan, it remains under the control of the manufacturer. Japan is the only country in the world that requires inpatient initiation. Moreover, psychiatrists need to be certified, and hospitals need to be certified by demonstrating agreement with other specialists to manage clozapine patients.^5^

Bachmann et al.^6^ published data on clozapine use in 17 countries in 2014: Finland had the highest usage with 189.2/100,000 persons, while Japan was reported as having an extremely low rate of 0.6/100,000. As of December 4, 2025, 25,214 patients had been registered in Japan's Clozaril Patient Monitoring Service including discontinued patients.^7^ Assuming a 35% discontinuation rate after 4 years of clozapine usage,^8^ we estimate that the number of patients currently using clozapine in Japan is approximately 10 per 100,000 persons, similar to 11 per 100,000 calculated from data from a 2024 study.^9^ According to the data provided by Novartis to the second author, there have been two deaths in Japan due to clozapine‐induced agranulocytosis since 2009, which provides a mortality rate of 0.008% (2/25,214).

A recent article has proposed that the significant number of fatal outcomes related to clozapine is not due to its use but its lack of use and consequent lack of prevention of suicide deaths in schizophrenia.^10^ The lifetime prevalence of suicide deaths was estimated by Palmer et al.^11^ to be around 5%. Many suicide deaths happened in the first 5 years of illness, and Palmer et al.^11^ described the rate among cohorts observed from a beginning illness point (5.6%) as more than three times that of cohorts observed from any other illness point (1.8%). Anyway, if we use a 5% lifetime rate and the data from the United States and Finland suggest that clozapine prevents 80% of suicide deaths,^12^ this will translate into clozapine's potential to save 4% of people with schizophrenia from suicide.^12^ Thus, if clozapine is prescribed to 10% of the worldwide population with schizophrenia, this would save 320,000 lives while costing 2400 deaths from agranulocytosis, based on a 0.03% recent US mortality rate.^10^ According to the FDA^2^ in 2023, there were 146,762 patients in the United States taking clozapine, which was 3.7% of US antipsychotic prescriptions. Hopefully, the elimination of the centralized database may help increase the limited use of clozapine in the United States. We can estimate^12^ that per each increase of 10,000 US prescriptions, 400 (4% of 10,000) suicide deaths will be prevented, but this may cost 3 (0.03% of 10,000) deaths from agranulocytosis.

The extremely low use of clozapine in Japan has probably resulted in substantial missed opportunities for suicide prevention among patients with schizophrenia. Assuming a schizophrenia prevalence of approximately 1% in Japan, this would correspond to roughly 1.2 million individuals with schizophrenia. Given an estimated lifetime suicide mortality risk of approximately 5%, the cumulative suicide burden among Japanese patients with schizophrenia may be substantial. Because TRS is estimated to affect approximately 30% of patients, many individuals potentially eligible for clozapine may not have received access to its anti‐suicidal benefits.

As a hypothetical example, if an additional 10,000 Japanese patients with TRS were treated with clozapine in the future, and if clozapine reduced suicide mortality by approximately 80% as suggested by international studies,^12^ approximately 400 suicide deaths might theoretically be prevented, assuming a 5% lifetime suicide mortality risk. In contrast, applying the observed Japanese agranulocytosis‐related mortality rate of 0.008% would correspond to approximately one agranulocytosis‐related death. Thus, this will provide a benefit‐to‐risk of 400 prevented suicide deaths versus 1 agranulocytosis death. This estimate may be conservative, since the 5% lifetime suicide mortality estimate was derived from schizophrenia populations overall, and suicide risk may potentially be higher among patients with TRS.

Following the FDA's change in June 2025, the European Medicines Agency (EMA) also issued a recommendation in August 2025 to relax clozapine blood monitoring requirements.^13^ Thus, Japan's hematological monitoring now substantially diverges from FDA and EMA recommendations. To promote the widespread use of clozapine in Japan, prevent suicide among patients with schizophrenia, and improve their quality of life, the urgent relaxation of Japan's clozapine monitoring system is necessary.

Finally, an additional caution regarding clozapine use in Japan is that the official titration schedule is very aggressive and the doses are too high for Japanese patients, leading to an extremely high number of clozapine‐induced inflammations.^14^

## AUTHOR CONTRIBUTIONS

Jose de Leon wrote the first draft of this article. All the co‐authors reviewed, corrected and approved the final version.

## CONFLICT OF INTEREST STATEMENT

In the last 3 years, the authors have had no conflicts of interest with commercial companies. Dr. Furakori is one of the editors‐in‐chief but was not involved in the editorial decisions about this article.

## ETHICS APPROVAL STATEMENT

N/A.

## PATIENT CONSENT STATEMENT

N/A.

## CLINICAL TRIAL REGISTRATION

N/A.

## Data Availability

Data sharing is not applicable to this article as no datasets were generated or analyzed during the current study.
